# Extraintestinal Pathogenic *Escherichia coli*: Beta-Lactam Antibiotic and Heavy Metal Resistance

**DOI:** 10.3390/antibiotics11030328

**Published:** 2022-03-01

**Authors:** Catia Longhi, Linda Maurizi, Antonietta Lucia Conte, Massimiliano Marazzato, Antonella Comanducci, Mauro Nicoletti, Carlo Zagaglia

**Affiliations:** 1 Dipartimento di Sanità Pubblica e Malattie Infettive, “Sapienza” Università di Roma, 00185 Rome, Italy; linda.maurizi@uniroma1.it (L.M.); antoniettalucia.conte@uniroma1.it (A.L.C.); massimiliano.marazzato@uniroma1.it (M.M.); antonella.comanducci@uniroma1.it (A.C.); carlo.zagaglia@uniroma1.it (C.Z.); 2 Dipartimento di Scienze Sperimentali e Cliniche, Università “G. D’Annunzio”, 66100 Chieti, Italy; mauro.nicoletti@uniroma1.it

**Keywords:** antibiotic resistance, metal tolerance, extraintestinal *Escherichia coli*, co-selection

## Abstract

Multiple-antibiotic-resistant (MAR) extra-intestinal pathogenic *Escherichia coli* (ExPEC) represents one of the most frequent causes of human nosocomial and community-acquired infections, whose eradication is of major concern for clinicians. ExPECs may inhabit indefinitely as commensal the gut of humans and other animals; from the intestine, they may move to colonize other tissues, where they are responsible for a number of diseases, including recurrent and uncomplicated UTIs, sepsis and neonatal meningitis. In the pre-antibiotic era, heavy metals were largely used as chemotherapeutics and/or as antimicrobials in human and animal healthcare. As with antibiotics, the global incidence of heavy metal tolerance in commensal, as well as in ExPEC, has increased following the ban in several countries of antibiotics as promoters of animal growth. Furthermore, it is believed that extensive bacterial exposure to heavy metals present in soil and water might have favored the increase in heavy-metal-tolerant microorganisms. The isolation of ExPEC strains with combined resistance to both antibiotics and heavy metals has become quite common and, remarkably, it has been recently shown that heavy metal resistance genes may co-select antibiotic-resistance genes. Despite their clinical relevance, the mechanisms underlining the development and spread of heavy metal tolerance have not been fully elucidated. The aim of this review is to present data regarding the development and spread of resistance to first-line antibiotics, such as beta-lactams, as well as tolerance to heavy metals in ExPEC strains.

## 1. Introduction

*Escherichia coli* is a harmless normal inhabitant of the gut of humans and warm-blooded animals. However, several pathogenic *E. coli* variants (pathotypes) endowed with the ability to cause intestinal (intestinal pathogenic *E. coli* or IPEC) or systemic infections (extra-intestinal pathogenic *E. coli* or ExPEC) have been described [1,2]. ExPEC strains are recognized as the most common Gram-negative human pathogens, globally responsible for the deaths of more than two million humans/year. ExPEC infections include urinary tract infections (UTI), hospital-acquired pneumonia, sepsis, surgical site infection, cholecystitis, cholangitis, peritonitis cellulitis osteomyelitis and arthritis. Moreover, *E. coli* is the leading cause of neonatal meningitis [3]. In this context, uropathogenic *E. coli* (UPEC) strains are the primary causes of community-acquired UTIs worldwide, with an estimated 40% of women and 12% of men, over the age of 18, who have to face to at least one symptomatic UTI episode during their lifetime, and with 27-48% of women suffering from recurrent UTIs [4,5,6]. Moreover, it has been suggested that the intestine could be the reservoir of UPECs, from which they can move to infect the urinary tract of the carrier, as well as that of other hosts, by the fecal–oral route and/or by sexual intercourse. In the intestine, ExPECs may stay asymptomatic even for a long time, while, under particular circumstances, they may gain entry into extra-intestinal tissues or districts by the expression of specific virulence determinants that allow them to colonize susceptible hosts [1,2,7,8]. Conversely, commensal *E. coli* strains rarely cause disease, except in immune-compromised hosts or, as in the case of peritonitis, when they may passively reach the peritoneal cavity through breaches in the gastrointestinal barrier. *E. coli* being endowed with notable genome plasticity, it is believed that pathogenic *E. coli* may have originated from commensals by the acquisition of specific virulence traits and by the loss or inactivation of house-keeping genes whose expression would negatively interfere with the new pathogenic lifestyle [1]. At the end of such complex recombination events, pathogenic mutants able to colonize new environmental niches may arise and spread the infections to other hosts. Furthermore, the newly generated pathogens have to fight against the host’s innate immune response, which may eliminate them, or mutants able to evade the host immune response may be selected [1,8]. Despite their clinical relevance, crucial aspects of ExPEC virulence (e.g., characterization of their reservoirs, routes of transmission, the mechanisms driving the onset of antibiotic- and heavy-metal-resistant mutants) remain to be fully elucidated. Understanding of these points will be of decisive relevance in order to develop new effective strategies aimed at counteracting or at least to limit the insurgence and the spread of these powerful pathogens. ExPECs harbor a variety of virulence-associated factors, including adhesins, toxins, iron acquisition systems, lipopolysaccharides, polysaccharide capsules, mobile genetic elements and the ability to manipulate the host innate immune response. Nevertheless, screening of clinical isolates for the simple carriage of virulence genes does not ensure correct identification of *E. coli* pathotypes since virulence-associated factors, found in ExPEC, are often found among commensal *E. coli* strains [4,9]. Due to biochemical similarities, the identification of the different pathotypes is not an easy task considering that specific, straightforward identification protocols are not available yet. Consequently, the identification of the different pathotypes is mainly based on taking into account the source of isolation, the carriage and expression of specific virulence-associated genes, the phylogenetic background and, when possible, their behavior in the experimental infection of suitable cellular/animal models. The introduction of molecular techniques, such as multiplex-PCR technology, the multilocus sequence type (MLST) and, more recently, phylogenetic comparison, extended to the whole bacterial genome, have highly contributed to providing new insights about this matter. Given the clinical importance of ExPEC and their propensity for the emergence of pandemic strains worldwide, surveillance systems are carried out to trace and prevent the global diffusion of pandemic serotypes. To this end, a simple and versatile method is phylogenetic analysis, which allows the classification of *E. coli* isolates into the A, B1, B2, C, D, E, F, G and clade I phylogroups [10,11,12]. Using this approach, commensal *E. coli* strains are placed mainly in the A or B1 phylogroups, while ExPEC strains are in the B2 group and, to a lesser extent, in group D. Strains of group E are related to those of group D, while group F is related to the main group, B2. Clones of *E. coli* strains, which are genetically diverse but phenotypically indistinguishable, are assigned to cryptic clade I [13]. Strains belonging to the same group may vary in the carriage and expression of relevant characteristics, such as antibiotic-resistance and virulence-associated genes. Today, multilocus sequence type (MLST) analysis is the gold standard, used to identify ExPEC strains as well as to evaluate their evolutionary relationships among different lineages. The method is based on PCR amplification and sequencing of internal DNA fragments of seven well-known *E. coli* house-keeping genes [14]. Differences in the DNA sequence are determined by comparison with specific databases of alleles. The alleles found at each locus define the allelic profile or sequence type (ST) of each isolate. The application range of the MLST technique is vast and it provides important information that may be of interest, such as environmental ExPEC reservoirs, whose identification might help the microbiological and clinical community to prevent the future insurgence and spread of high-risk pandemic lineages within the community setting. In the last decade, the development of next-generation sequencing (NGS) techniques made the access to the whole-genome sequences (WGSs) of microorganisms fast and cost-effective. The huge amount of genomic data produced by NGS clearly determines the entire genomic content of a single microorganism, permitting us to delineate the whole gene repertoire carried by microbial taxa and to extend the genomic comparisons among strains to the whole-genome level. In this context, the classification of *E. coli* strains, based on WGS, gradually integrates the seven loci MLST-based ST-typing and related classification systems, allowing deeper knowledge of the origin, evolution and spread of these strains. 

Despite the huge diversity of ExPEC STs, it has been recently reported that ExPEC ST12, ST69, ST73, ST95, ST127 and ST131 are the predominant lineages causing extraintestinal infections worldwide [15,16,17]. Among these lineages, it has been shown that ST69 is mainly associated with resistance to trimethoprim–sulfamethoxazole [18], while the UPEC pandemic ST131 lineage appears to be associated with fluoroquinolone resistance and with β-lactam resistance by extended-spectrum β-lactamase (ESBL) enzymes. In addition, it has been proposed that ST131 may have significantly contributed to the global dissemination of cephalosporin and fluoroquinolone resistance [19,20].

Conversely, the finding that ST95, ST73 and ST127 are fully antibiotic-susceptible indicated that factors other than antibiotic resistance might be responsible for the global distribution of these ST lineages. Infections due to multiple-antibiotic-resistant (MAR) pathogenic bacteria are steadily increasing worldwide, both in hospital and community settings, rendering always more difficult their control. Consequently, the number of therapy failures is also increasing, together with increased hospitalization, morbidity/mortality and societal costs. With respect to the recent past, this condition is worsened considering that we are in the so-called “post-antibiotic era”, when the discovery of new, effective antibacterial drugs is dramatically low. In this context, the scarcity of new, effective antibiotics, together with expanding antibiotic resistance, has renewed the interest of the scientific community in heavy metals as substitutes for antibiotics and as adjuvants for the efficacy of already existing ones [9].

## 2. Resistance to Antibiotics and Heavy Metals

### 2.1. Antibiotic Resistance

The mechanisms by which bacteria become antibiotic-resistant are diverse, complex and not completely understood. In this regard, commensal *E. coli* strains, especially those inhabiting the intestines of humans and warm-blooded animals, have been shown to play a pivotal role in the onset and diffusion of antibiotic-resistance genes [21,22]. The high intestinal bacterial density, together with antibiotic exposure, due to global indiscriminate and, in many cases, inappropriate use of antibiotics, have likely led to the selection and spread of antibiotic-resistance mutants among commensal *E. coli* strains, which are considered a primary reservoir of antibiotic-resistance genes. Moreover, the remarkable *E. coli* genome plasticity may have favored the acquisition of virulence-associated determinants that may be transmitted to bacteria of the same and/or of related species [8,23].

The results of epidemiological surveys indicate that 20–45% of ExPEC isolates were resistant to first-line antibiotics including beta-lactams, fluoroquinolones and trimethoprim–sulfamethoxazole [24,25,26,27], so that the management of patients suffering from MAR ExPEC infections has become more complicated.

The fluoroquinolones are a family of broad-spectrum antibiotics used since the late 1980s for the treatment of severe infections, especially UTIs, although long-term therapy with fluoroquinolones is linked to serious adverse effects. Consequently, the U.S. Food and Drug Administration recommends the restricted use of these antibiotics to UTIs untreatable with commonly used antibiotics. Nevertheless, fluoroquinolones continue to be widely prescribed in several countries [16,17,18,19,20] and the incidence of resistant *E. coli* is continuously increasing. To resist to the action of fluoroquinolones (the inhibition of topoisomerase enzymes, DNA gyrase and topoisomerase IV), *E. coli* may become resistant by mutations that alter the target enzymes, by reducing the entrance of the antibiotic into the cell or by activating efflux systems, which extrude the drug from the cell. Aminoglycosides are a family of bactericidal broad-spectrum antibiotics that may also act in synergy with other antibiotics. Despite the adverse side effects due to the prolonged use of these antibiotics, several members of this family were intensively used in the past, until they were replaced in the 1980s with cephalosporins, carbapenems and fluoroquinolones. The mechanism of action of aminoglycosides targets the binding to the aminoacyl-tRNA recognition site (A-site) of the 16S rRNA of 30S ribosome, leading to the inhibition of polypeptide synthesis and subsequent cell death. Resistance to aminoglycosides may occur based on several mechanisms, namely (i) enzymatic modification and inactivation of the aminoglycosides, mediated by aminoglycoside acetyltransferases, nucleotidyltransferases or phosphotransferases, both in Gram-positive and -negative bacteria; (ii) increased efflux of the antibiotic outside the cell; (iii) decreased permeability; and iv) modifications of the 30S ribosomal subunit that alter the binding of the aminoglycosides [28,29,30,31,32,33].

### 2.2. Heavy Metal Resistance

In the pre-antibiotic era, heavy metals were largely used as chemotherapeutics and/or as antimicrobials in human and animal healthcare, and some heavy metals are still in use today in medicine and agriculture settings [34]. As with antibiotics, resistance to heavy metals has recently increased, and ExPECs presenting combined resistance to both antibiotics and heavy metals are commonly isolated [9,13,16,17,18,19,35,36]. This finding is of particular concern considering that heavy metals have been associated with the co-selection of antibiotic resistance genes and that, thanks to mobile genetic elements, heavy metal resistance genes are transmitted to microorganisms of the same or of related species [37,38]. Although the efficacy of metal preparations is controversial, nevertheless, several heavy metals are still in use in agriculture and aquaculture (i) as organic/inorganic fertilizers; (ii) as preservatives in animal food production and (iii) as biocides and antimicrobials in cases of antibiotic failure [39]. Due to their elevated toxicity, some of the treatments with metals such as mercury and arsenic/antimony have now been eliminated or greatly limited in several countries [40,41]. Since they are chemically stable elements, heavy metals accumulate in soil and water and exercise a strong selective pressure that may trigger the onset of metal-resistant mutants. Thus, decontamination of metal-polluted zones appears to be an inevitable step to limit the development and diffusion of both antibiotic and metal resistance genes [42]. This is of particular concern also considering that the environmental metal pollution is remarkably more diffused than antibiotic pollution. According to what is reported above, the strong association between metal exposure, metal- and antibiotic-resistance genes led the European Scientific Committee for Emerging and Newly Identified Health Risks to warn about bacterial exposure to heavy metals, which may boost the onset and the spread of antibiotic-resistance genes [35]. Apart from their importance in co-selection, heavy metals also play a role in the regulatory mechanisms governing complex aspects of the bacterial physiology, such as the two-component CpxA/CpxR system of Gram-negative bacteria, which senses and responds to stress stimuli, such as those caused by cell-envelope-targeting drugs and heavy metals. Once activated, the CpxA/CpxR system activates the transcription of 11 out of 28 Cu-inducible genes whose expression is also required to transform Cu-susceptible microorganisms into Cu-tolerant derivatives [43]. Furthermore, as well as counteracting antibiotic activity, bacteria have to face the potential lethal effects of poisonous oxygen derivatives (molecular oxygen does not react with cell components, but its derivatives are toxic) such as hydrogen peroxide and superoxide anions, which directly undermine the metabolic activities of enzymes co-factored with iron and flavins. Consequently, when bacteria accumulate substantial reactive oxygen insults, coming from environmental or cellular sources, growth is inhibited. Although hydrogen peroxide and superoxide anions do not oxidize DNA directly, these molecules feed directly or indirectly the generation of the highly reactive hydroxyl radicals, which damages the bacterial chromosome [44]. Characteristically, three reactive oxygen species (ROS) are considered, i.e., superoxide anions (O^2−^), hydrogen peroxide (H_2_O_2_) and hydroxyl radicals (HO•), though other reactive and abundant molecules may exist. Several common oxidants derived from molecular oxygen are found in the environment or even produced by the cellular metabolism. To meet this challenge, *E. coli* activates complex mechanisms to resist the accumulation of these radicals. The activity of the sigma factor protein family is linked to bacterial stress responses. In *E. coli*, activation of RpoE is required for maintaining the integrity of periplasmic and outer membrane compartments; it is regulated by Zn and also responsible for the transition to Zn- and Cu-tolerance phenotypes [45]. Again, Cu has also been shown to regulate the expression of the oxidative stress-responsive regulatory gene *soxR*, whose encoded SoxS protein regulates the expression of the AcrAB efflux pump [46]. Lastly, it has been recently reported that exposure to sub-inhibitory concentrations of heavy metals, such as Cu, Ag, Cr and Zn, promotes the conjugative transfer of antibiotic-resistance genes. This finding highlights even more the urgent need to develop new, effective strategies to control the environmental diffusion of these metal pollutants [9,47,48,49,50].

## 3. Resistance to Beta-Lactams

Beta-lactams (comprising penicillins, cephalosporins, carbapenems and monobactams) are one of classes of antibiotics most successful for the treatment of bacterial infections. However, over the years, their efficacy has been considerably challenged by the rapid spread worldwide of bacteria resistant to β-lactams.

Beta-lactam antibiotics are bactericidal agents towards actively replicating bacteria; they affect the formation of the bacterial cell wall by covalently binding to the penicillin-binding proteins (PBPs) that are involved in the terminal steps of peptidoglycan cross-linking in both Gram-negative and Gram-positive bacteria. Penicillin G (benzylpenicillin) was the first beta-lactam to be clinically used, mostly to treat streptococcal infections, towards which it shows high activity. Penicillin V (phenoxymethylpenicillin) is another naturally occurring penicillin whose oral formulation is used therapeutically and prophylactically for mild to moderate infections in pediatric patients [51].

The rise of resistant strains quickly followed the introduction of penicillins for clinical use. The most common beta-lactam resistance mechanism found in Gram-negative bacteria is the production of beta-lactamase periplasmic enzymes that hydrolyze the beta-lactam ring. The rapid increase in the incidence of clinically relevant beta-lactamase-producing strains prompted the search for penicillins endowed with increased stability towards these enzymes. In the 1950s, the discovery of the beta-lactamase-stable cephalosporin C introduced new opportunities for the development of a number of novel cephalosporins very active against major beta-lactamase-producing pathogens of medical interest. Several products were introduced into clinical practice, although it was quickly evident that, as with penicillins, the use of cephalosporins in medical practice would have led to the rapid onset of resistant mutants [52].

Beta-lactamases represent the most common mechanism of resistance to beta-lactams in Gram-negative bacteria. AmpC-type β-lactamases and extended-spectrum β-lactamases (ESBLs) represent the two groups of hydrolytic enzymes mainly involved in cephalosporin resistance. The term AmpC defines a class of enzymes that belong to the molecular class C (of the Ambler’s classification system), whereas ESBLs belong to class A and are able to hydrolyze later-generation cephalosporines. A serine residue is present within the active site of both AmpC and ESBL enzymes, though their protein sequences are remarkably different [8,53,54,55]. The class B, which groups metallo-beta lactamases (MBLs) and zinc(II)-dependent enzymes that can accommodate β-lactams in their active site, is able to hydrolyze almost all β-lactams, including carbapenems [56]. Recently, the global dissemination of Gram-negative bacteria harboring plasmid-encoded MBLs (e.g., the New-Delhi metallo-β-lactamase (NDM-1)) has increased the clinical relevance of this class of enzymes. The carriage of plasmid-encoded MBLs seems to be the reason for the high level of expression of this class of enzymes, as well their rapid dissemination [54,55]. The extensive (mis)use of ß-lactams in the recent past in human and animal settings is believed to have favored the selection of beta-lactam resistance worldwide [57]. The search for novel broad-spectrum beta-lactamase inhibitors that may work against many problematic beta-lactamases, such as cephalosporinases and serine-based carbapenemases, which severely limit their therapeutic use, is underway. Carbapenems are a class of highly effective, broad-spectrum antibiotics commonly used for the treatment of severe or high-risk bacterial infections by Gram-positive and Gram-negative microorganisms. As a result, they are often used as “last-line agents” in patients who are gravely ill or suspected of harboring resistant bacteria. The recent emergence of multidrug-resistant pathogens seriously threatens this class of lifesaving drugs. As with other beta-lactams, carbapenems bind to critical PBPs, disrupting the growth and structural integrity of bacterial cell walls [58,59,60].

The antibacterial efficacy of this class of antibiotics lies mainly in the high affinity for penicillin-binding proteins (PBPs) and in their excellent stability against β-lactamases [60]; carbapenems enter Gram-negative bacteria through outer membrane porins and, after crossing the periplasmic space, they “permanently” acylate the PBPs. Furthermore, carbapenems are strong inducers of chromosomal β-lactamases, which may lead to antagonism to penicillins and cephalosporins. Initially, it was thought that bacteria producing carbapenamases circulated mainly among long-term-hospitalized patients; nowadays, the global dissemination of carbapenemase-producing Gram-negative environmental bacteria highlights the need to develop new, efficient strategies to limit the incidence of carbapenem-resistant microorganisms [60,61].

### 3.1. AmpC Beta-Lactamases

In *E. coli*, the AmpC beta-lactamase-encoding gene can be either chromosomal (c*ampC*) or plasmid-associated (p*ampC*). c*ampC* is expressed at low levels since it is under the control of a weak promoter and a strong attenuator [4]. The main feature of cAmpC is represented by its variable level of expression that may be constitutive or inducible by several β-lactams, resulting in different resistance phenotypes. When c*ampC* is constitutively expressed, it hydrolyzes penicillins, most cephalosporins, cephamycins and monobactams, but not the fourth-generation cephalosporins and carbapenems. Furthermore, AmpC β-lactamases are not inhibited by “classical” β-lactamase inhibitors [62]. The regulation of ca*mpC* expression in *E. coli* is considerably different from other *Enterobacteriaceae*. *E. coli* lacks *amp*R, and thus c*ampC* expression cannot be inducible. However, various mutations in the c*ampC* promoter/attenuator region may result in overexpression of c*ampC* [63]. It is generally accepted that p*ampC* likely originated by the horizontal gene transfer of a c*ampC* gene of *Enterobacteriaceae* [64]. A surveillance study conducted in Canada indicated that CMY-2-producing *E. coli* could be considered a pathogen whose principal target appears to be the urogenital tract of older women [65]. Failure to detect AmpC-overproducing strains may hamper the clinical management of patients subjected to cephalosporin-based therapy [63,66]. In addition, it has been reported that AmpC overproduction together with porin mutations may reduce susceptibility to carbapenems [67].

### 3.2. Extended-Spectrum Beta-Lactamases

First described in 1983, the extended-spectrum beta-lactamases (ESBLs) belong to the family of the so-called “newer” beta-lactamases. From the late 1990s, ExPEC strains producing ESBLs begun to emerge globally. ESBLs are able to hydrolize penicillins, cephalosporins and monobactams, but not carbapenems and cephamycins, and their hydrolytic activity is subject to the inhibitory effect of β-lactamase inhibitors. Recently, an ESBL variant, the CTX-M β-lactamase, has become the most common and globally widespread ESBL type [4,68]. Epidemiological surveys have shown an alarming link between CTX-M-15 and resistance to TMP-SMZ, tetracycline, gentamicin, tobramycin and ciprofloxacin [24]. It has been suggested that the global spread of the pandemic UPEC strain of sequence type ST131 may have been favored by the worldwide insurgence of CTX-15-producing *E. coli*. Furthermore, by comparing *E. coli* strains producing CTX-M with *E. coli* strains encoding other ESBLs, it was found that the former were more antibiotic-resistant and more commonly linked to cases of community-acquired UTIs. Thus, ESBLs are considered the predominant source of *E. coli* resistance against the new generation of cephalosporins, and CTX-M-15-producing *E. coli* ST131 is the principal source of community-acquired UTIs [4,18,69].

### 3.3. Metallo-Beta-Lactamases

Metallo-β-lactamases (MBLs) are a family of enzymes that require Zn^2+^ for operating. MBLs probably originated from commensal and/or environmental bacteria and, from them, horizontally transferred to microorganisms of the same or related species [70]. According to the beta-lactamase sequence-based Ambler’s classification system, class B represents the zinc-dependent MBLs, whereas class A, C and D are serine beta-lactamases [71]. Class B enzymes are of particular concern since they present a broad spectrum of activity against almost all β-lactam antibiotics. Based on sequence similarities and the employment of zinc in catalysis, MBLs have been grouped into three subclasses, namely B1, B2 and B3, which differ in the active site residues, metal content requirement and substrate specificity [72,73].

The type of metallo-β-lactamase found in *Klebsiella pneumoniae* and *E. coli* strains, which was isolated from a Swedish patient previously hospitalized at New Delhi, and named NDM-1, is particularly relevant: it is able to hydrolyze a variety of beta-lactams, including penicillins, cephalosporins and carbapenems, except monobactams (aztreonam), and is inhibited by EDTA [74,75,76]. Since then, several NDM-1 variants were identified in *Enterobacteriaceae*, in *Vibrionaceae* and in other non-fermenter Gram-negative bacteria. It is thought that NDM-1 probably originated from a very rare genetic fusion event between two previously known antibiotic-resistance genes [75]. Moreover, the majority of NDM-producing bacteria were also found to be resistant to various classes of antibiotics, including aminoglycosides and fluoroquinolones, further reducing the therapeutic options [4,77]. In this context, it appears evident that MBLs are key players for antibiotic resistance. The majority of NDMs identified so far belong to subclass B1. B1 beta-lactamases require one or two Zn^2+^ ions for catalysis. Among MBLs, the most clinically significant B1 beta-lactamases are NDM, VIM, IMP, SPM and CcrA [73]. Of these, the genes encoding IMP, VIM and NDM are largely plasmid-encoded and thus may be horizontally transmitted to bacteria of the same or related species, contributing to the inter/intra-species genetic variability of these strains [77]. A number of variants of NDMs have been described in Gram-negative pathogens, including ExPECs and *E. coli* of animal origin [21]. Interestingly, the first MBL of the subclass B1 to be described, in 1966, was the BcII beta-lactamase isolated from *Bacillus cereus*, a spore-forming environmental bacterium [72].

Concerning MBLs of subclass B2, they are mainly chromosomally encoded and require only one Zn^2+^ ion to operate. MBLs of subclass B2 are strict carbapenemases acting poorly on penicillins and cephalosporins [78]. Regarding MBLs of subclass B3, they can operate either with one or two Zn^2+^ ions. MBLs of subclass B3 present a broad substrate spectrum of activity and, remarkably, they share only nine conserved residues with the other MBLs [79].

It is reasonable to believe that NDMs might have contributed to the worldwide spread and success of the pandemic ST131 *E. coli* strain [4,80]. Regardless, NDM-producing bacteria have to be considered particularly risky (i) because of the lack of available standardized phenotypic tests; and (ii) because of the scarcity of new antibiotics effective for the treatment of these virulent MAR *E. coli* strains. Accordingly, there is agreement in considering a priority the development of new, efficient strategies to counteract CTX-M- and NDM-producing *E. coli* strains [81].

## 4. Metal Resistance

The availability of antibiotics was the endpoint for the wide antimicrobial usage of heavy metal preparations both in medicine and veterinary science. The rapid emergence and diffusion of MAR pathogens worldwide, together with the scarcity of new, effective antibiotics, has led the medical community to reconsider the use of some heavy metals against infections untreatable with conventional antibiotic-based therapies. Heavy metals are naturally present in the environment. We will consider heavy metals that are extensively utilized in agriculture and as additives in feed or preservatives in food, and in medicine.

### 4.1. Resistance to Arsenic

In the environment, microorganisms are continuously exposed to heavy metals, some of which are taken up as essential nutrients, whereas others (e.g., mercury, lead, cadmium, arsenic and silver) are toxic for a variety of organisms, including bacteria [82]. Among heavy metals endowed with toxic activity, arsenic (As) is of particular concern, mainly because of its use in agriculture settings. Arsenic is a ubiquitous element present primarily in natural geological as well as in anthropogenic sources (e.g., herbicides, pesticides, wood preservatives, animal feeds and semiconductors). Despite its elevated toxicity, in the past, As has been extensively used in human and animal healthcare. In nature, As is present in soil and in groundwater, where it is found in the oxidation states of arsenite As(III), the most dangerous As form, and arsenate As(V) [83]. Although there are a number of methods suitable to rescue As-contaminated areas, none is devoid of serious drawbacks. Actually, the use of engineered bacteria able to absorb and transform As(III) into the less toxic As(V) seems to be very promising to resolve this problem. However, since As oxidation is influenced by pH changes, it is possible that As(III)-decontaminated areas may revert to the previous As(III)-contaminated one. Now, since As(V) is also toxic, researchers have agreed that the best strategy to detoxify As-contaminated areas is to reduce As(III) to the more stable and insoluble form, As(0) [84].

As represents a relevant health threat for human populations living near contaminated areas since long-term As exposure (mainly through the consumption of contaminated drinking water and food) has been associated with several severe diseases, including cardiovascular and peripheral vascular diseases, neurological disorders, diabetes mellitus and various forms of cancer. For these reasons, As ranks first on the Superfund List of dangerous compounds (http://www.atsdr.cdc.gov/spl/, accessed on 6 November 2021) and its use has been largely restricted or even banned in several countries [85,86]. To escape from As toxicity, nearly every organism has evolved export strategies (the *ars* operon), mainly directed at extruding As(III) outside the cell and thus reducing its intracellular accumulation. Regarding *Enterobacteriaceae*, *ars* operons have been found in *Serratia marcescens*, *Yersinia* spp. *Klebsiella* spp. and *Salmonella enterica* [82]. In *E. coli*, the *ars* operon may be either chromosomal or plasmid-encoded. The chromosomal *ars* operon (arsRBC) codes for three proteins, namely (i) ArsC, an arsenate-reductase that oxidizes intracellular As(III) to As(V), which is then exported from the cells by an energy-dependent efflux process; (ii) ArsB, an arsenate-permease that confers As resistance by pumping As(V) outside the cell; and (iii) ArsR, an arsenic-inducible repressor regulating *ars* operon expression. Conversely, more complex appears the expression of the plasmid-encoded *ars*RDABC operon, where arsA codes for an ATPase that binds to the ArsB permease and to ArsD, which appears to function as a second trans-acting repressor that prevents excess transcription [87,88,89,90]. Interestingly, Sütterlin et al., 2018 [91], by investigating the susceptibility to heavy metals of a cohort of 186 putative ExPEC isolated from urine samples of patients hospitalized in Sweden, Germany and Spain, found the *ars* operon present only in all non-B2 phylogenetic groups, which have previously been associated with the environment and commensalism in both humans and animals, while all B2 clades lacked a functional *ars* operon and consequentially were susceptible to sodium arsenite. The absence of a functional *ars* operon appears to be characteristic of the putative ExPEC strains examined; thus, it seems to lead to a survival disadvantage for those strains; on the contrary, bacteria might benefit in some way from the lack of a functional *ars* operon. Further investigation is needed to understand why arsenic resistance in *E. coli* strains appears prevalent among *E. coli* phylogroups associated with the environment rather than among *E. coli* pathotypes. These findings highlight the importance of better knowledge of the phylogenetic relationships among *E. coli* strains, whose elucidation might contribute to a better understanding of the mechanisms that drive the resistance and selection of ExPEC in human and veterinary settings [91].

### 4.2. Resistance to Silver

Although categorized among the list of toxic metals, silver (Ag) does not pose significant risks to human health, whereas it is highly toxic, even at low concentrations, for most microorganisms [37,92]. The silver cation (Ag^+^) has a long history of use as an antimicrobial agent and it is still used in several bacterial infections, especially in the prevention and treatment of burns and chronic wound infections. Moreover, silver-impregnated dressings and antimicrobial coatings are used in infection management and in the stimulation of wound healing. In addition, silver is used in dental amalgam and silver-impregnated medical devices, such as catheters and heart valves. The widespread and uncontrolled use of silver may result in increasing the rate of bacterial resistance to silver-containing compounds, where silver-resistant bacteria are as problematic as antibiotic-resistant ones [93,94].

Hence, there is a fear that the increasing development and spread of Ag-tolerant microorganisms may compromise the therapeutic utility of this valuable element. 

Initially, Ag-tolerant bacterial species were found mainly among environmental bacteria isolated from areas highly polluted by heavy metals. Likely due to the extensive use of heavy metals in livestock farm activities and the lack of effective decontamination measures, the incidence of metal-tolerant bacteria has increased together with the co-selection of antibiotic-resistance genes. The risk of increasing antibiotic resistance has led several laboratories to investigate the mechanisms underlying the onset of heavy metal tolerance in a number of bacterial species [95].

In this context, Gram-positive bacteria do not appear to represent a problem since there is no evidence to date of the development of Ag^+^ tolerance in Gram-positive pathogens, such as *Staphylococcus aureus*. On the other hand, low prevalence of Ag^+^ resistance in Gram-negative pathogens exposed to silver nitrate has been reported [91]. 

Silver exerts its toxic activity by oxidizing sensitive cellular thiol groups and by inducing the production of reactive oxygen species (ROS), which damage the cell membrane. To protect themselves from the oxidative stress and to become Ag-tolerant, Gram-negative bacteria utilize the *sil* operon [46]. In *E. coli*, resistance to Ag^+^ mainly depends on a combination of periplasmic Ag^+^ sequestration and subsequent efflux outside the cells [96]. Silver resistance can arise by mutation (endogenous-acquired) or by horizontal gene transfer (exogeneous-acquired). Endogenous-acquired resistance to silver in Gram-negative bacteria can arise from the expression of the chromosomal Cus system and from the loss of outer membrane porins, whereas exogenous-acquired silver resistance is due to the acquisition of a plasmid-encoded *sil* system. A *sil* operon was first described in 1975 on plasmid pMG101 (an IncHI2 incompatibility group plasmid, which carries several antibiotic-resistance genes as well as tolerance to mercury, tellurite and silver; present in an epidemic strain of *Salmonella enterica* serovar Typhimurium [97]). To evaluate the incidence of cryptic Ag^+^ resistance, Elkrewi et al., 2017 [96] analyzed a collection of 444 Gram-negative clinical pathogens by plating bacteria onto agar plates containing AgNO_3_. Using this approach, previous results have been confirmed indicating that overt Ag^+^ resistance is not so common, while cryptic *sil* operons are found in the majority of *Enterobacter* spp. and of *Klebsiella* spp. strains [96]. Only one isolate out 135 *E. coli* strains tested (0.7%) was found to be Ag^+^-resistant. The finding that none of the isolates tested displayed overt Ag^+^-tolerance clearly indicated that cryptic *sil* operons are common in Gram-negative pathogens such as *Enterobacter* and *Klebsiella* spp. Regarding the *sil* operon, it comprises nine open reading frames (ORFs) (*silE*, *silS*, *silR*, *silC*, *silF*, *silB*, *silA*, *ORF105* and *silP*) defining three transcriptional units [94]. Either endogenous or exogenous, *sil*-mediated resistance mainly consists of preventing intracellular silver accumulation. To this end, SilE, a small periplasmic silver-binding protein, binds silver at the cell surface, presenting the first line of defense against silver toxicity, together with the two-component silver-responsive transcriptional regulatory system silRS. This system controls the expression of the silver efflux ATPase *silP* gene, of the tripartite silCBA silver effluxer and of *silF*, which is a periplasmic silver chaperone. Endogenous silver resistance is mainly due to porin loss and overexpression of the Cus system, which contributes to silver efflux and to limited Ag accumulation inside the cell [94,98]. Further knowledge regarding the mechanisms involved in the development and regulation of *sil* operon expression is a prerequisite to understand the meaning of the carriage of a cryptic *sil* operon in Gram-negative bacteria.

### 4.3. Resistance to Cadmium

Heavy metal contamination can impact soil ecosystems, resulting in significant loss of soil quality by altering biological processes catalyzed by microorganisms. Microorganisms are highly adaptable to extreme environmental conditions such as those characterized by heavy metal contamination. Cadmium (Cd) is a non-essential, non-biodegradable, highly diffused metal element, extremely toxic for all organisms. When Cd enters into a susceptible host cell, it accumulates in the cytoplasm, altering the activity of several enzymes and directly attacking nuclear DNA [99,100]. The worldwide presence of Cd in nature is considered to be mainly due to ungoverned industrial and anthropological activities, such as corrosive reagents, as stabilizers in the synthesis of PVC products, mining, electroplating, as stabilizers of plastics, manufacturing batteries, alloy pigments and high-phosphate fertilizers [101,102].

Heavy metal pollution poses many risks to human health considering that human consumption of cadmium-contaminated food, water or air has been associated with the development of severe kidney, lung, liver, bone and reproductive system diseases. Moreover, according to the International Agency for Research on Cancer, Cd is now designated as a human carcinogen [103,104]. However, some microbes survive well in Cd-polluted environments as they have evolved various metal resistance mechanisms [105]. Bacterial exposure to Cd may give rise to Cd-tolerant mutants as well as to the co-selection of antibiotic-resistance genes [106]. Thus, as Cd pollution is a primary source of health concern for human and animal health, measures have been taken to decontaminate and conserve the environment by reducing this type of heavy metal pollution. To this end, although several investigations have been conducted in order to develop suitable tools to decontaminate aqueous metal-polluted environments, so far, none has been found to be completely satisfactory. Actually, a quite promising approach for bioremediation appears to be that centered on the development of engineered microorganisms [105]. To cope with the damaging effects of the intracellular accumulation of Cd^2+^ in the cell, *E. coli* strains have developed a variety of resistance mechanisms, including efflux transport of Cd^2+^ outside the cell and intracellular sequestration by precipitation by metal-binding proteins, such as metallothionein and other thiol-containing compounds. Czc is one of the best-characterized metal efflux systems and confers resistance against cadmium, zinc and cobalt in many Gram-negative bacteria, including *E. coli* [107]. In *E. coli*, the cadmium efflux pump operon *czcCBA* consists of three structural genes whose products form a complex efflux pump. Of these, CzcA is the chemiosmotic cation/H^+^ antiporter, CzcB is the membrane fusion protein, and CzcC is the outer membrane protein [108]. Furthermore, chelation of metal ions by intracellular metallothioneins (MTs) is another strategy used by *E. coli* to survive under Cd toxicity. MTs are a family of small polypeptides with a high percentage of cysteine residues and metal-binding sites, which bind Cd ions, thus contributing to the maintenance of homeostasis and to the induction of Cd tolerance. MTs also play a role in other cellular processes, including DNA damage repair, the metabolism of metallo-drugs, responses to stress conditions and scavenging of reactive oxygen species [109]. In particular, the transition from Cd susceptibility to tolerance appears to be regulated by the coordinated expression of some chromosomal genes, including the *zntA* gene, which encodes for the Pb^2+^-, Zn^2+^- and Cd^2+^-transporting ATPases, and the *capB* gene, whose expression also enhances *E. coli* Cd resistance [100,110]. In addition, the plasmid-associated *czcABC* operon and the *cnr* system are also thought to be involved in Cd^2+^ tolerance. Moreover, by generating *E. coli* B strain BL21(D3) mutants highly resistant to Cd, two other genes were identified, namely *htpX* and *gor*. At present, it is impossible to exclude that other genes related to Cd resistance may exist in the *E. coli* genome. A better understanding of the mechanism underlining Cd tolerance is needed to improve the development of new, effective tools to be used in order to reduce the presence of this toxic element in the environment [100].

### 4.4. Resistance to Copper and Zinc

Antibiotic and heavy metal supplementation has been extensively used to promote growth and animal health. Alongside antibiotics, heavy metals have been used in livestock production for a long time. However, the negative impact of the environmental release of these substances on the development and spread of antibiotic- as well as of heavy-metal-resistant microorganisms has recently led to their ban in several countries. Copper (Cu) and zinc (Zn) are two ubiquitous essential trace elements whose principal role is to ensure proper cellular functions [111]. Regarding Cu, this element has been shown to be necessary for the human body because its major function includes fetal growth and early postnatal development, hemoglobin synthesis, maturation of the connective tissues and inflammatory processes [112]. Cu supplementation is used to prevent Cu-deficiency syndromes. To ensure that the necessary amount of Cu is available for absorption in agriculture settings, the concentrations of Cu added to feed are, in general, higher than necessary, highly contributing to increased Cu pollution as well as the selective pressure towards environmental microorganisms [95]. Needed to sustain life, excess Cu is extremely toxic, with Cu(I) being more toxic than Cu(II), which damages iron–sulfur clusters and catalyzes the production of reactive oxygen species [113]. Organisms have had to develop strategies to deal with the toxic effects of Cu to cells. However, it was recently reported that, at therapeutic concentrations, Cu might also exert a selection pressure on the gut microbiota that may lead to the development of Cu-tolerant mutants. To avoid toxicity, bacteria have evolved effective strategies to limit the amount of Cu accumulation in the cell through the synthesis of specific metal transport systems that balance the influx and efflux of metals to and from the cell [95,114]. Nevertheless, exposure to metals also selects for the development of metal-resistant strains, which, in turn, will increase the risk of co-selection of antibiotic-resistant genes [95].

The antimicrobial nature of copper has been recognized since ancient times so that when, in 2006, the UE banned the use of antibiotics in animal husbandry, considered the primary reservoirs of virulence and antibiotic-resistance genes, much interest turned towards heavy metals as alternative antibiotics [115]. A relevant characteristic of Cu toxicity lies in its ability to undergo redox cycling between Cu(II), the less toxic oxidized cupric form, and Cu(I), the toxic cuprous reduced form. Under aerobic conditions, this redox cycling generates highly reactive hydroxyl radicals that damage biomolecules, such as proteins, lipids and DNA. In *E. coli*, at physiological Cu levels, these fluctuations are under the control of two chromosomal operons, the copper translocating P-type ATPase CopA system, and the central components in copper homeostasis, the multi-copper oxidase CueO and the multi-component copper transport system CusCFBA, which remove the excess of Cu(I), thus protecting the periplasmic space from copper-induced toxicity. As soon as Cu enters the *E. coli* cytoplasm, cytosolic chaperones bind and transport Cu to copper-requiring enzymes, such as cytochrome oxidase, superoxide dismutase and lysyl oxidase, as well as to specific efflux systems, such as members of the P_1B-1_-ATPase subfamily, including Cu(I) transporters and the Cpx-ATPase [95,116]. The chromosomally encoded P_1B-1_-ATPases are involved in the removal of excesses of cytoplasmic Cu out of the cell. Of these, the best-characterized ones are those of the copper homeostasis systems, including *copA*, which encodes the P_1B_-ATPase *copA*. *copA* is part of the CueR regulon, where the repressor CueR binds Cu(I) and regulates the expression of *copA* and *cueO* [95,117]. While *copA* expels Cu(I) from the cytoplasm, its presence in the periplasm may cause damage to the cell. To cope with this, bacteria utilize *cueO*, a periplasmic multicopper oxidase enzyme, which oxidizes Cu(I) to Cu(II). Genes encoding homologs of *copA* and *cueO* are present. Other than to be chromosomally encoded, Cu-resistant determinants have been found also on *E. coli* plasmids linked to the presence of the *pco* system, which encodes the two-component regulatory system PcoRS [95,118,119]. Zn is the second most abundant transition metal in seawater and in humans. It is an essential trace element required to fulfill the dietary requirements of all organisms. Zn is considerably less toxic than redox-active metals such as copper and is more soluble than iron. Zn is widely used in the pig farming industry in order to overcome problems during weaning, including infections caused by pathogenic *E. coli* [120,121,122]. Remarkably, Zn serves as a cofactor in a variety of enzymes, as well as in several regulatory proteins. Several families of integral membrane proteins transport Zn(II), moving it across membranes into and outside cells [95]. The precise mechanisms used by *E. coli* to sense, store and/or incorporate Zn(II) as a cofactor have to be fully elucidated. A common assumption is that Zn(II)-requiring enzymes and transcription factors acquire this essential element from a cytosolic pool, assumed to be 10^−16^ M in free Zn(II) [45,123,124]. Several Zn(II)-responsive transcription factors are known to mediate zinc homeostasis by sensing changes in the cytosolic level of free Zn(II) [95]. Bacteria exposed to elevated concentrations of Zn(II), such as those encountered in contaminated soil and water, and in feed additives in agriculture, may lead to the insurgence of Zn-tolerant mutants. Recent studies conducted with piglets fed with high concentrations of Zn reported a rise in the proportion of multidrug-resistant *E. coli* in the guts of the animals [115,125,126]. These findings have generated doubts regarding the convenience of the use of this element in agriculture, since Zn-associated co-selection with antibiotic-resistance genes has been demonstrated [115,127,128,129]. Accordingly, withdrawal of veterinary products containing Zn has been highly recommended in the EU states [95]. In *E. coli*, Zn binds to around 10% of all bacterial proteins, contributing to proper 3D structure and catalysis [95]. Interestingly, the antioxidant enzyme copper-zinc-superoxide dismutase (CuZn-SOD) is unique in requiring both metals for its catalytic activity [115]. As for copper, *E. coli* resistance to Zn mainly depends on the ability to control the cytoplasmic concentration of Zn(II) by uptake and efflux-specific systems [130]. In *E. coli*, the Zn(II) regulators ZntR and Zur are activated in the presence of very low concentrations of free Zn(II). At least three systems (ABC transporter, ZIP transporter and phosphate-bound uptake) are known to be involved in bacterial zinc uptake, while at least four systems (P_1B_-type ATPases, CDF transporters, 2-TM-GxN transporters and CBA efflux systems), either chromosomally or plasmid-encoded, have been found to be involved in transporting Zn(II) outside the cell. Under Zn-limiting conditions, *E. coli* utilizes the ABC (ATP binding cassette) transporter ZnuABC operon, where ZnuA is a zinc-binding protein, ZnuB spans the cytoplasmic membrane and forms the channel used to transport Zn(II) into the cytoplasm, and ZnuC is bound to the cytoplasmic site on ZnuB, providing the energy required for Zn(II) transport via ATP hydrolysis [131]. Thus, since several metal-resistance genes, including those encoding for Cu and Zn tolerance, have been involved in the co-selection of antibiotic-resistance genes, the limited use of these elements and/or decontamination of polluted soil and groundwater sites appear to be essential tasks to limit the onset and diffusion of multiple pathogenic, antibiotic-resistant bacteria. Furthermore, along with their role in co-selection, heavy metals may form complexes with several classes of antibiotics, whose effects may vary from the inactivation and/or reduction of antibiotic activity, decrease in metal availability, increase in impermeability-based resistance mechanisms or perturbation of metal-bound proteins [113]. These findings strongly support the belief that heavy metal tolerance may be considered an important virulence factor of pathogenic microorganisms [132].

## 5. Metal Nanoparticles

The incidence of MAR pathogens is a serious health problem and their eradication has become a very difficult or even impossible task to achieve with the traditional antimicrobial therapy, thus leading to an increase in morbidity and mortality. Consequently, several alternative strategies to combat these MAR pathogens have been proposed; metal nanoparticles (NPs) are considered one of the most promising alternatives. Actually, NPs are increasingly used as a delivery system for medical products, including antibiotics, or directly as antibacterial agents. Although the exact mechanism through which NPs exert their antimicrobial activity is not completely understood, metal and metal oxide NPs have been shown to have many useful features, such as to modify both the toxicity and the antibacterial activity of antibiotics; to damage the bacterial outer membrane and/or cell wall; to favor the interaction between intra- and extracellular components and ions; to produce reactive oxygen species that damage bacterial structures; to inhibit DNA synthesis; to inhibit enzyme activity [133,134].

## 6. Conclusions

Extra-intestinal pathogenic *E. coli* (ExPEC) strains represent one of the most frequent causes of human infections, resulting in massive direct medical and social costs and approximately two million human deaths/year. ExPEC strains comprise several pathogenic lineages, of which only a few are responsible for the vast majority of infections. The emergence and the worldwide spread of multiple-antibiotic-resistant (MAR) ExPEC strains that we are witnessing today are of particular concern due to the shortage of new antibiotics that are effective against these MAR pathogens and to the limited success of the measures that have been put in place to control their development and propagation (e.g., the sparing use of antibiotics in order to prolong their effectiveness; the ban of the use of antibiotic as animal growth promoters). Although the mechanism(s) underlining the development and the global spread of MAR ExPEC strains have not been completely elucidated yet, the widespread and very often unnecessary use of antibiotics in clinical and agriculture settings is considered to be the leading cause of this condition. Similarly to antibiotics, resistance to heavy metals has recently increased, likely due to the long-lasting strong selective pressure exercised by the environmental anthropogenic levels of these substances (unlike antibiotics, heavy metals are not subject to degradation). As a result, *E. coli* strains, pathogenic or not, presenting a combination of heavy metals and antibiotic-resistance genes are now frequently isolated. The rise of heavy metal resistance is of great concern because (i) heavy metal resistance determinants appear to play an important role in the selection and diffusion of antibiotic-resistance genes through co-carriage and/or co-selection of antibiotic and metals resistance genes; and (ii) heavy metals are still in use in medicine to treat a number of bacterial infections, so heavy-metal-tolerant mutants may limit their potential therapeutic use. In conclusion, this review highlights recent aspects of antibiotic and heavy metal resistance. Future research will be needed to develop effective strategies addressed at limiting the impact of MAR pathogens on human and animal health.

## Data Availability

Not applicable.
